# The Marine Natural Product Pseudopterosin Blocks Cytokine Release of Triple-Negative Breast Cancer and Monocytic Leukemia Cells by Inhibiting NF-κB Signaling

**DOI:** 10.3390/md15090262

**Published:** 2017-08-23

**Authors:** Julia Sperlich, Russell Kerr, Nicole Teusch

**Affiliations:** 1 Bio-Pharmaceutical Chemistry & Molecular Pharmacology, Faculty of Applied Natural Sciences, Technische Hochschule Koeln, Chempark, 51368 Leverkusen, Germany; julia.sperlich@th-koeln.de; 2 Department of Chemistry, and Department of Biomedical Sciences, Atlantic Veterinary College, University of Prince Edward Island, Charlottetown, PE C1A 4P3, Canada; rkerr@upei.ca

**Keywords:** pseudopterosin, NF-κB, p65, inflammation, tumor microenvironment, breast cancer, cytokine release, IL-6, TNFα, MCP-1, glucocorticoid receptor

## Abstract

Pseudopterosins are a group of marine diterpene glycosides which possess an array of biological activities including anti-inflammatory effects. However, despite the striking in vivo anti-inflammatory potential, the underlying in vitro molecular mode of action remains elusive. To date, few studies have examined pseudopterosin effects on cancer cells. However, to our knowledge, no studies have explored their ability to block cytokine release in breast cancer cells and the respective bidirectional communication with associated immune cells. The present work demonstrates that pseudopterosins have the ability to block the key inflammatory signaling pathway nuclear factor κB (NF-κB) by inhibiting the phosphorylation of p65 and IκB (nuclear factor of kappa light polypeptide gene enhancer in B-cells inhibitor) in leukemia and in breast cancer cells, respectively. Blockade of NF-κB leads to subsequent reduction of the production of the pro-inflammatory cytokines interleukin-6 (IL-6), tumor necrosis factor alpha (TNFα) and monocyte chemotactic protein 1 (MCP-1). Furthermore, pseudopterosin treatment reduces cytokine expression induced by conditioned media in both cell lines investigated. Interestingly, the presence of pseudopterosins induces a nuclear translocation of the glucocorticoid receptor. When knocking down the glucocorticoid receptor, the natural product loses the ability to block cytokine expression. Thus, we hypothesize that pseudopterosins inhibit NF-κB through activation of the glucocorticoid receptor in triple negative breast cancer.

## 1. Introduction

Cancer represents one of the diseases with the highest unmet medical need, causing the second highest incidence of death after cardiovascular diseases in industrialized countries. Among the different types of malignant tumors, breast cancer is the leading cause of cancer mortalities in women worldwide [1]. Classification of breast cancer subtypes is based on the expression of progesterone receptor (PR), estrogen receptor (ER) and/or human epidermal growth factor receptor (HER2). Accordingly, the breast cancer subtype expressing none of these three receptors, the so-called triple-negative breast cancer (TNBC), represents the most aggressive form with currently no targeted therapy available and a significantly reduced overall survival rate [2,3]. Thus, development of innovative and more effective therapies is urgently needed.

Marine organisms represent a vast source of biologically active compounds with a highly unexploited potential for innovative drug development [4]. For instance, the soft coral *Antillogorgia elisabethae* (formerly *Pseudopterogorgia elisabethae*) has been reported to produce at least 31 different secondary metabolites, most of which have not been pharmacologically unexplored [5]. Amongst others, the pseudopterosin family displays a broad spectrum of biological activities, including anti-inflammatory [6,7,8], analgesic [6,9,10], wound-healing [7,8] and neuromodulatory [11] activity. Moreover, pseudopterosins have shown anti-inflammatory efficacy in phase II clinical trials [12,13] and represent the first commercially licensed marine natural product for use in cosmetic skin care [7,11]. Intriguingly, in vivo assays revealed a higher efficacy of pseudopterosins against topically induced inflammation than the marketed drug indomethacin [6]. Despite the striking in vivo pharmacological effect [6,10,14] and the application in cosmetic products [7,11] the underlying in vitro mechanism of action of the anti-inflammatory potential of pseudopterosins remains elusive. The potential of pseudopterosin A (PsA) has been described as spreading across different intracellular mechanisms ranging from inhibition of phospholipase A2 [10], altering calcium release [15], and inducing cytotoxicity in cancer cells [16]. To our knowledge, no studies have explored the potential of pseudopterosins as a novel immune modulatory agent in breast cancer.

A key factor in regulating inflammatory responses is the transcription factor nuclear factor κB (NF-κB) that acts by controlling expression of cytokines and chemokines. Activation can be triggered by various factors including pro-inflammatory cytokines, growth factors, hormones, oxidative stress, viral infections or DNA-damaging agents [17,18,19,20]. Pathogen-associated-molecular-patterns (PAMPs) such as lipopolysaccharides (LPS) and tumor necrosis factor alpha (TNFα) are ligands of different receptors, both triggering activation of the NF-κB-controlled immune response [21,22,23]. The NF-κB family consists of five functionally conserved members in mammalian cells, including RELA (nuclear factor NF-kappa-B subunit p65), RELB (nuclear factor NF-kappa-B subunit p60), c-REL, NF-κB1 (p105 and p50) and NF-κB2 (p100 and p52) [24]. The specific activation of NF-κB in the innate and adaptive immune defense is opposed by constitutive NF-κB expression in various tumor types. Constitutive activation of NF-κB could be confirmed in cancer in general, and in breast cancer in particular, supporting overall tumor progression, drug resistance, invasiveness, epithelial-to-mesenchymal-transition (EMT) and the promotion of hormone-independent growth [17,25,26,27,28]. Elevated NF-kB activity has been observed in both primary human breast cancer tissues and breast cancer cell lines. Furthermore, a recent study assigned a key role of NF-kB in disrupting important microenvironmental cues necessary for tissue organization [29]. The tumor microenvironment (TME) encompasses a complex interplay between tumor cells and tumor associated immune cells. Tumor associated macrophages (TAM) play a crucial role in cancer progression [30]. Tumor associated macrophages produce high amounts of cytokines such as interleukin-6 (IL-6), interleukin-8 (IL-8), monocyte chemotactic protein 1 (MCP-1) and tumor necrosis factor alpha (TNFα) to alter the tumor progression in different ways. IL-6 promotes tumor proliferation, IL-8 leads to neovascularization, growth, angiogenesis and metastasis, and TNFα affects necrosis, invasion and metastasis [26,27]. Moreover, MCP-1 overexpression correlates with histological grade and low level differentiation in breast tumors [31].

The glucocorticoid receptor alpha (GR) has been investigated in different clinical studies as a putative pharmacological target for the treatment of breast cancer [32,33,34]. Interestingly, there is evidence that NF-κB and GR can physically interact and heterodimerize in breast cancer [35]. By binding other transcription factors such as NF-κB or AP-1, GR can either transactivate or suppress its target genes [1]. Agonism of glucocorticoids (GC) can block migration, invasion and angiogenesis via down-regulation of IL-6 and IL-8 and has been reported to induce drug sensitivity. Furthermore, GC activation induces apoptosis in lymphoid cancer and MCF-7 breast cancer cells [36,37,38]. However, due to high variability in its expression frequency, divergent cellular functions of GR have been described [2]. Herein, we describe inhibitory capabilities of a mixture of pseudopterosins on the NF-κB signaling pathway and its target genes, the cytokines, in monocytic leukemia and in triple negative breast cancer cells (TNBC) presumably by agonizing the glucocorticoid receptor α. Moreover, our study ascribes the efficient cytokine blockade in the context of bidirectional tumor-immune-cell communication to pseudopterosin treatment.

## 2. Results

### 2.1. Pseudopterosin Reduces Cytokine Release by Inhibition of NF-κB Signaling

**Figure 1:**
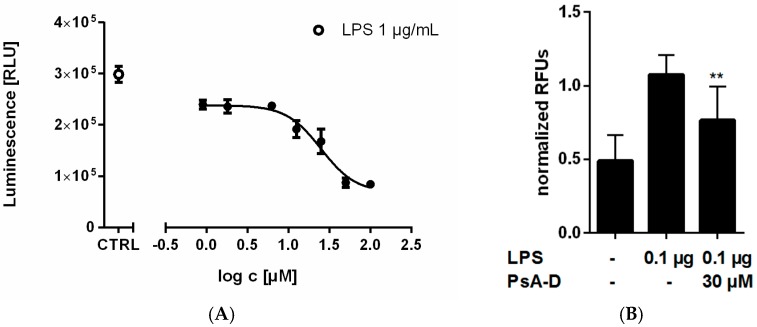
Nuclear factor κB (NF-κB) inhibition in lipopolysaccharide (LPS)-stimulated stable NF-κB-MDA-MB-231 and THP-1 monocytic leukemia cells. (**A**) Dose–response curve of pseudopterosin (PsA-D) on LPS stimulated NF-κB-MDA-MB-231 cells expressing a luciferase reporter gene which is under the control of a NF-κB CMV (cytomegalovirus) promotor. Luminescence intensity correlates proportionally with NF-κB activation. The solid circle represents NF-κB induction in the presence of 1 µg/mL LPS (positive control). PsA-D treatment was performed for 20 min in a bisecting titration followed by 1 µg/mL LPS for 1 h. IC_50_ value of 24.4 µM of pseudopterosin was calculated from three independent experiments; (**B**) Inhibition of NF-κB upon pseudopterosin treatment in THP-1 monocytic leukemia cells (ELISA). Cells were incubated with PsA-D for 20 min followed by LPS treatment. Pseudopterosin decreased NF-κB activation significantly. RLU = relative luminescence units; RFU = relative fluorescence units. Two stars represent a significance of *p* < 0.05. Error bars were calculated using standard error of the mean (+SEM); *n* = 3.

Pseudopterosins have been described as anti-inflammatory agents with an unknown in vitro mechanism of action. To explore intracellular signaling pathways following pseudopterosin treatment, we investigated the influence of an extract mixture containing four different pseudopterosin derivatives (PsA-D) on the key inflammatory signaling pathway NF-κB. For this purpose, we generated a stable cell line based on the triple negative breast cancer cell line MDA-MB-231 (subsequently named NF-κB-MDA-MB-231) (see Section 4.2, Stable Cell Line Generation). MDA-MB-231 cells display a high level of toll-like-receptor 4 (TLR4) [39] which can activate NF-κB signaling via its ligand LPS [40]. Interestingly, increasing amounts of pseudopterosin inhibited LPS-induced NF-κB activation in NF-κB-MDA-MB-231 breast cancer cells in a concentration-dependent manner (Figure 1A) with an IC_50_ value of 24.4 µM. Additional studies revealed that pseudopterosin also reduced NF-κB activation initiated by other stimuli including TNFα (Figure S1). Moreover, addition of 30 µM of pseudopterosin in monocytic THP-1 cells led to a 1.65-fold inhibition of NF-κB-dependent luciferase activity (Figure 1B).

As multiple pro-inflammatory cytokines such as IL-1, IL-6 and TNFα represent target genes of NF-κB [41,42,43], we investigated the effect of PsA-D on pro-inflammatory cytokine release.

Analyzing a subset of six different cytokines simultaneously, in THP-1 cells incubated with 1 µg/mL LPS led to a significant secretion of IL-6, TNFα and MCP-1 compared with unstimulated control (23-fold induction of IL-6, 33-fold induction of TNFα and 24-fold increase of MCP-1; Table 1), but not IL-1β, IL-12 or IL-4 (data not shown). Compared to THP-1 cells, MDA-MB-231 breast cancer cells displayed a higher basic level of IL-6 and MCP-1. Upon LPS stimulation, we confirmed a 3-fold increase of IL-6, a 15-fold induction of TNFα and a 5-fold increase of MCP- 1 in MDA-MB-231 cells (Table 1). In contrast, no induction of IL-1β or IL-4 could be observed in the triple negative breast cancer cells (data not shown). In both cell lines investigated, PsA-D incubation was able to induce a significant blockade of cytokine secretion: In THP-1 monocytic leukemia cells pseudopterosin reduced TNFα release by at least 47%, blocked IL-6 release by 50% and MCP-1 release by 73%. In MDA-MB-231 breast cancer cells incubated with PsA-D led to a reduction of MCP-1 by 85%, a decrease of TNFα release by 75%, and a decrease of IL-6 by 38%.

**Figure 2:**
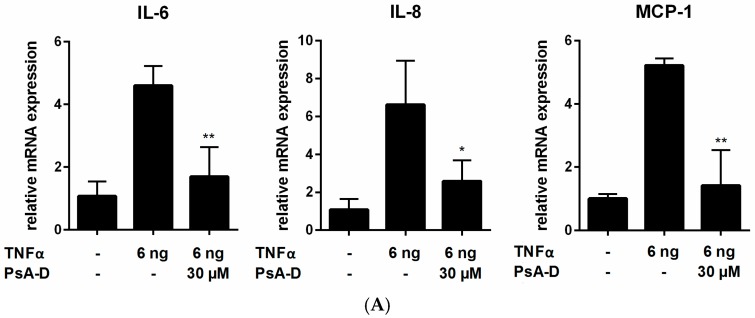

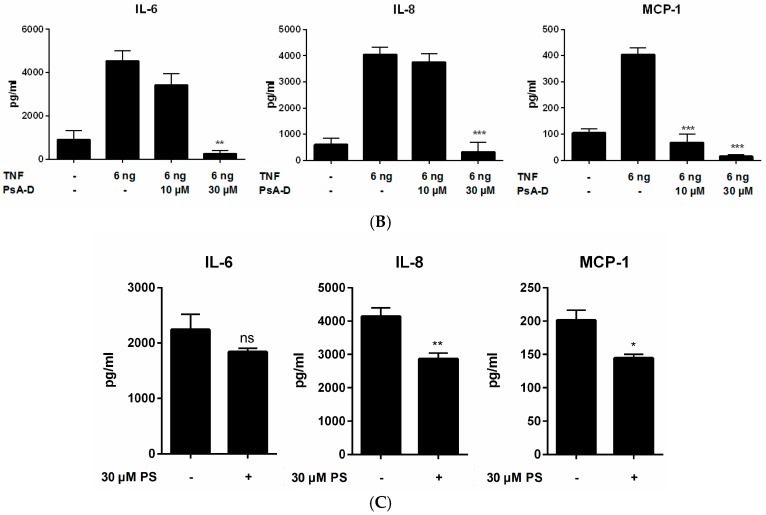
Inhibition of cytokine expression (**A**) and secretion (**B**) after TNFα stimulation and inhibition of endogenous cytokine secretion (**C**) in MDA-MB-231 triple-negative breast cancer (TNBC). (**A**) MDA-MB-231 cells were treated with 30 µM of PsA-D for 20 min followed by 6 ng/mL of TNFα for 5 h; (**B**) Various concentrations of PsA-D were incubated for 20 min followed by TNFα treatment for 24 h; (**C**) MDA-MB-231 cells were treated with 30 µM of PsA-D and cytokine secretion was measured 24 h thereafter. Error bars were calculated using +SEM; *n* = 3. *p*-Values are calculated against TNFα. Three stars represent a significance of *p* < 0.001, two stars *p* < 0.01, one star *p* < 0.05 and “ns.” means not significant.

As the NF-κB signaling pathway can be activated with different stimuli including LPS, TNFα or pathogen-associated molecular patterns (PAMPs) [18,44,45], we utilized TNFα, the ligand of the TNFα receptor 1 (TNFR1) [23,46], to induce NF-κB signaling independent of TLR4. As expected, stimulation with TNFα increased the expression levels of the investigated cytokines in MDA-MB-231 breast cancer cells significantly compared to unstimulated control (IL-6 4-fold, IL-8 6-fold, MCP-1 5-fold) (Figure 2A). It is noteworthy that pseudopterosin blocked the expression of all cytokines investigated, however, statistical significance was only noted for IL-6 and MCP-1 (IL-6 2.7-fold induction, MCP-1 3.7-fold induction).

Secretion of cytokines is stimulated after TNFα treatment (IL-6 4540 ± 329 pg/mL, IL-8 4047 ± 196 pg/mL, MCP-1 4048 ± 18 pg/mL) (Figure 2B). Cytokine amounts declined in the triple negative breast cancer cells in a concentration-dependent manner upon pseudopterosin treatment (at a PsA-D concentration of 30 µM: 18-fold decrease of IL-6, 12-fold reduction of IL-8 and a 26-fold decrease of MCP-1). Significant inhibition at a concentration of 10 µM of PsA-D could be achieved for MCP-1 (6-fold decrease of MCP-1 release compared to untreated control).

It is noteworthy that irrespective of exogenous cytokine stimulation via LPS or TNFα, pseudopterosins are able to significantly reduce endogenous release of at least two cytokines in the MDA-MB-231 triple negative breast cancer cells (IL-6 1.2-fold, IL-8 1.4-fold, MCP-1 1.4-fold) (Figure 2C). Moreover, additional investigation demonstrates that the reported inhibitory effect of PsA-D on cytokine release can be assigned to other triple negative cell lines (Table S1).

### 2.2. Pseudopterosin Blocks Bidirectional Communication

**Figure 3:**
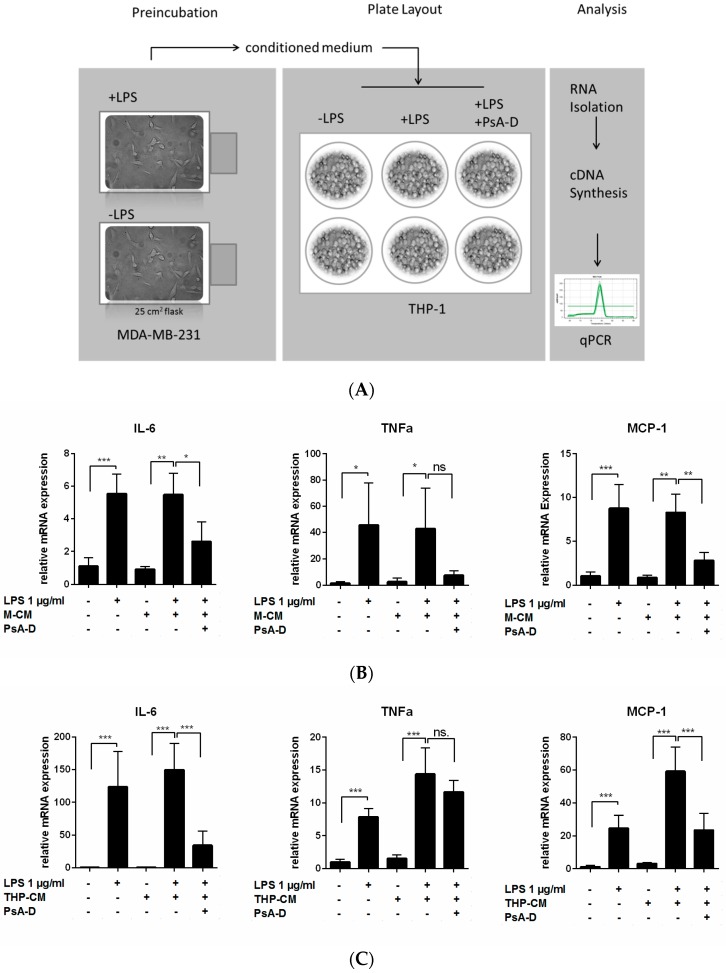
Blockage of bidirectional communication between THP-1 monocytic leukemia and MDA-MB-231 TNBC. (**A**) Process scheme of producing tumor conditioned medium. THP-1 or MDA-MB-231 cells were cultured in 25 cm^2^ flasks and treated with 1 µg/mL LPS for 24 h. Medium was collected and centrifuged. After sterile filtration, tumor conditioned medium was added to seeded cells in 6-well plates. (**B**) MDA-MB-231 conditioned medium (M-CM) or (**C**) THP-1 conditioned medium (THP-CM) was added to the adversary cells. RNA was isolated for further analysis in real-time PCR. Error bars were calculated using +SEM. *p*-Values of three stars represent a significance of *p* < 0.001, two stars *p* < 0.01, one star *p* < 0.05 and “ns.” means not significant.

To explore whether pseudopterosins have the ability to inhibit the bidirectional communication between immune cells and tumor cells, we designed an experimental set-up imitating inter-cell communication within the tumor microenvironment (Figure 3A). As shown, stimulation by LPS leads to the production of secondary metabolites including cytokines and the subsequent secretion into the surrounding “conditioned medium” (CM). Medium containing cytokines released by MDA-MB-231 cells represents the so called “MDA-MB-231 conditioned medium” (M-CM; Figure 3B), whereas medium encompassing cytokines secreted by THP-1 cells referred to as “THP-1 conditioned medium” (THP-CM; Figure 3C). Both conditioned media were used in independent experiments to stimulate the respective opposite cell line. Treatment with unstimulated conditioned medium did not influence cytokine expression in any of the investigated cell lines. However, incubation of THP-1 leukemia cells with stimulated M-CM induced a significant cytokine expression in THP-1 cells (8-fold increase of IL-6, 18-fold induction of TNFα and nearly 13-fold in MCP-1 expression). Furthermore, the triple negative breast cancer cell line MDA-MB-231 induced expression of IL-6, TNFα and MCP-1 in the presence of stimulated THP-CM (IL-6 induction 177-fold, TNFα induction nearly 10-fold and MCP-1 induction nearly 19-fold).

Notably, pseudopterosin treatment was able to block cytokine expression induced by conditioned media in both leukemia cells and in triple negative breast cancer cells. In THP-1 cells stimulated with M-CM, a 2-fold reduction of IL-6 expression and a 3-fold reduction of MCP-1 expression was noted following pseudopterosin treatment. Also, MDA-MB-231 cells stimulated with THP-CM displayed a 4-fold increase in IL-6 and a 2.5-fold increase in MCP-1 expression. In conclusion, our data demonstrate that PsA-D is able to significantly decrease expression of the cytokines IL-6 and MCP-1 after stimulation with pre-conditioned medium in monocytes and breast cancer cells, respectively.

**Figure 4:**
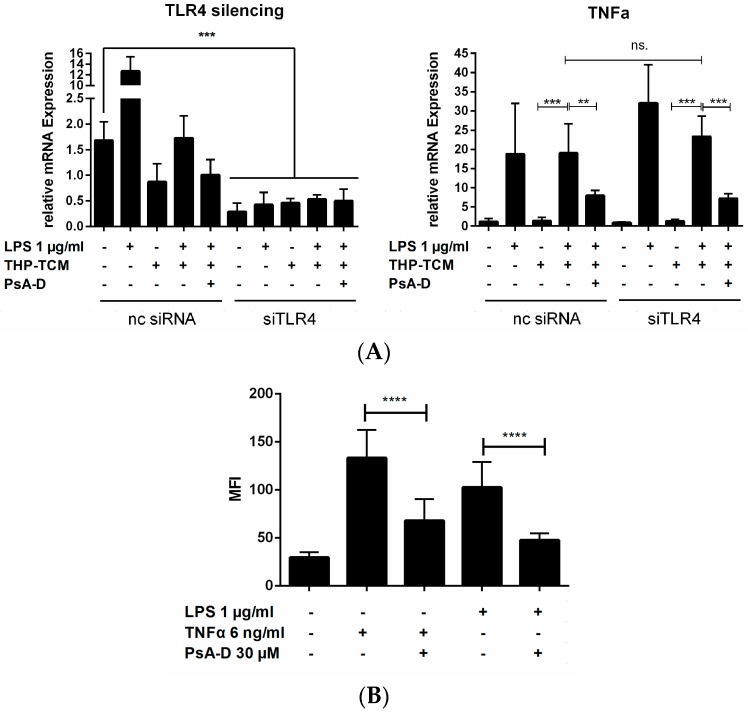
PsA-D induced NF-κB inhibition is toll-like-receptor-4 (TLR4)-independent. (**A**) MDA-MB-231 cells were seeded in 6-well plates and incubated for 24 h. Transfection with 2 µM siRNA was done with Lipofectamine3000 following the manufacturer’s protocol. After 24 h, cells were first treated with 30 µM PsA-D before and following treatment with THP-CM for 5 h. After another 24 h of incubation, cells were harvested and lysed for RNA isolation in preparation for realtime PCR. Knock-down efficiency of TLR4 was about 50%. PsA-D blocked TNFα expression independent of TLR4 expression; (**B**) MDA-MB-231 cells were stimulated either with 1 µg/mL LPS or with 6 ng/mL TNFα following 20 min treatment of PsA-D. P65 phosphorylation was measured after 24 h of treatment. Error bars were calculated using +SEM. *p*-Values of four stars show a significance of *p* < 0.0001, three stars *p* < 0.001, two stars *p* < 0.01 and “ns.” means not significant.

To exclusively ascribe the demonstrated cytokine expression patterns to the pre-treatment with the respective conditioned medium, we subjected MDA-MB-231 cells to a knock-down of the TLR4 receptor (siRNA-TLR4 (siTLR4) transfected cells) (Figure 4A). As a control, we transfected non-coding silencing RNA (nc siRNA). A 50% TLR4 knock down was achieved. Compared to a nc siRNA control, siTLR4 transfection did not influence TNFα expression level upon pseudopterosin treatment. Monitoring the p65 phosphorylation with TNFα and LPS in parallel experiments we confirmed a 2-fold reduction of phosphorylation after pseudopterosin treatment independent of the stimulus (Figure 4B). In conclusion, PsA-D induced cytokine blockade and p65 phosphorylation in triple negative breast cancer cells does not dependent on TLR4.

### 2.3. Pseudopterosin Inhibits NF-κB through Activation of the Glucocorticoid Receptor

**Figure 5:**
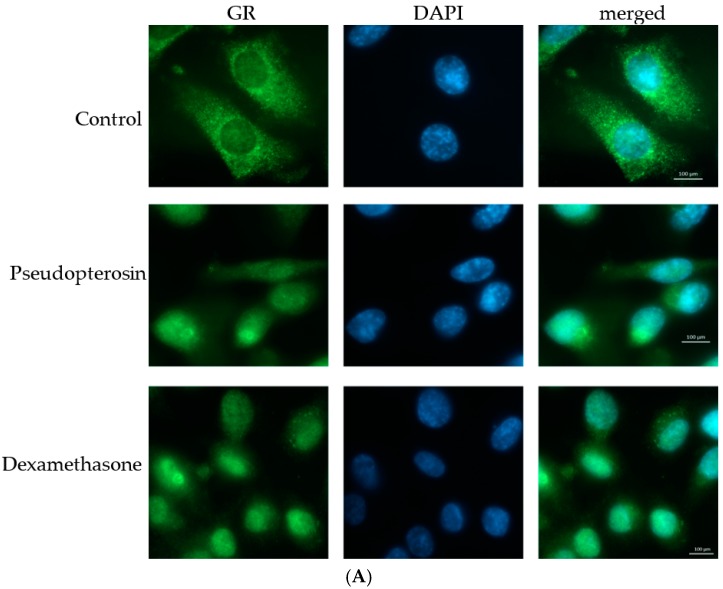

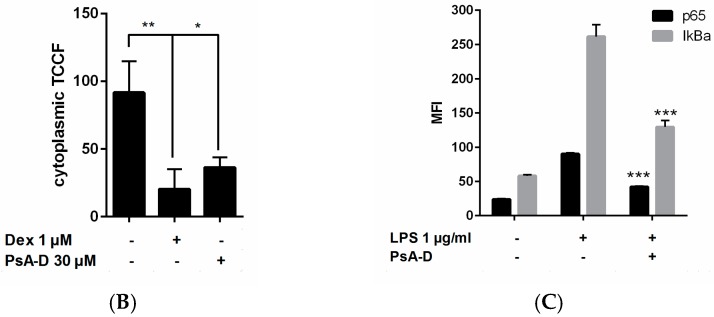
Pseudopterosin-induced activation of glucocorticoid receptor alpha (GR) translocation into the nucleus is accompanied by inhibition of phosphorylation of p65. (**A**) PsA-D was added at a concentration of 30 µM and dexamethasone at 1 µM in MDA-MB-231 cells. Cell nuclei were stained with 3 µM 4′,6-Diamidin-2-phenylindol (DAPI; blue channel). GR is shown in green. The right column shows merged channels; (**B**) Quantification of immunofluorescence staining shows cytoplasmic total corrected cell fluorescence (TCCF). TCCF was calculated as described in methods section. Cytoplasmic TCCF was calculated after following formula: TCCF GFP–TCCF DAPI. Cytoplasmic staining reduced significantly after dexamethasone (Dex) or PsA-D treatment; (**C**) Phosphorylation of p65 and IκBα induced by LPS was investigated in the absence or presence of PsA-D with an incubation time of 20 min on MDA-MB-231 breast cancer cells. *p*-Values of three stars show a significance of *p* < 0.001, two stars of *p* < 0.01 and one star of *p* < 0.05; +SEM; *n* = 30. MFI = median fluorescence intensity.

Our data show for the first time that the underlying in vitro mechanism of the well described anti-inflammatory response of pseudopterosin might be ascribed to inhibition of the NF-κB pathway. To further explore putative molecular pharmacological targets of pseudopterosins, we started to investigate the influence of the natural product on glucocorticoid signaling. NF-κB and glucocorticoid receptor α (GR) display opposed functions in regulating immune and inflammatory responses. Moreover, both transcription factors have been described as transcriptional antagonists [36]. Thus, we investigated the interaction of pseudopterosin with GR. To evaluate transactivation of GR in the presence of PsA-D on the whole cell level, we used immunofluorescent staining of GR in MDA-MB-231 cells incubating the cells with dexamethasone, serving as a positive control, or PsA-D (Figure 5A). Untreated cells displayed an even GR distribution within the cytosol, whereas the nucleus did not show any GR localization. As expected, upon dexamethasone treatment the GR staining revealed a complete translocation of the receptor to the nucleus in breast cancer cells. Interestingly, the presence of pseudopterosin induced a comparable nuclear translocation of the GR. Quantification of the respective fluorescence intensities using the software ImageJ confirmed a significant GR translocation to the nucleus after dexamethasone treatment (4.5-fold reduction of cytoplasmic total corrected cell fluorescence (TCCF) compared to control) and pseudopterosin treatment (2.5-fold reduction of cytoplasmic total corrected cell fluorescence (TCCF) compared to control, Figure 5B). Accordingly, PsA-D inhibited phosphorylation of p65 and IκBα significantly compared to LPS stimulation (Figure 5C) or compared to stimulation with TNFα (Figure S2) (2-fold inhibition, respectively).

**Figure 6:**
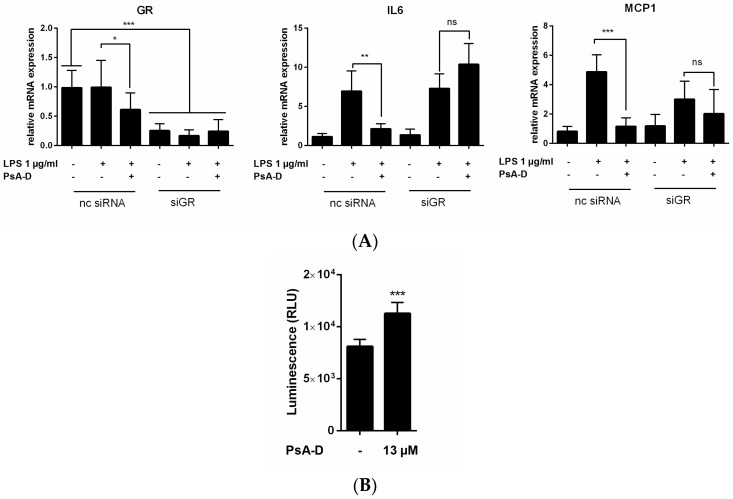
Pseudopterosin as a low molecular weight agonist of GR. (**A**) MDA-MB-231 cells were seeded in 6-well plates and transfected with 2 µM siRNA with the Nucleofector^®^ 2b device using the manufacturer’s protocol. After 24 h, cells first were treated with 30 µM PsA-D for 20 min and subsequently with 1 µg/mL LPS for 24 h. After another 24 h of incubation, cells were harvested and lysed for RNA isolation as preparation for further real-time PCR analysis; (**B**) Cells were seeded following manufacturer’s instructions. Reporter cells stably expressing a luciferase under the control of a human GR promotor were activated upon pseudopterosin treatment. Error bars were calculated using +SEM; (**A**) *n* = 3; (**B**) *n* = 2. *p*-Values of three stars show a significance of *p* < 0.001, two stars *p* < 0.01, one star *p* < 0.05 and “ns.” means not significant.

Moreover, to confirm GR as a putative pharmacological target of pseudopterosin we performed a glucocorticoid receptor α knock-down in MDA-MB-231 cells. In this context, we transfected cells with siRNA of GR (siGR, Figure 6) with non-coding siRNA (nc siRNA) serving as a negative control. A 60% knock-down of GR was achieved. Treatment with negative control nc siRNA revealed that unaltered GR expression resulted in cytokine expression level after LPS stimulation comparable to previous results (Table 1). Furthermore, as demonstrated earlier, pseudopterosin inhibited IL-6 (3-fold) and MCP-1 (nearly 4-fold) significantly in the presence of GR. However, when knocking down GR, pseudopterosin lost the ability to block IL-6 or MCP-1 expression, respectively. To finally confirm glucocorticoid receptor α as a potential pharmaceutical target for pseudopterosin, we used a reporter gene assay expressing a luciferase under the control of a human GR promotor (Figure 6B). In line with our previous findings, pseudopterosin induced a significant increase in expression of human GR. In conclusion, the described inhibitory effect of pseudopterosin on cytokine expression and release in triple negative breast cancer is putatively ascribed to agonism of glucocorticoid receptor α.

## 3. Discussion

Though their mechanism of action remains unknown, pseudopterosins have been demonstrated as anti-inflammatory [6,7,8], analgesic [6,9,10], wound-healing [7,8], anti-microbial [47,48], and anti-cancer agents [16]. In our work we were able to illuminate a novel molecular mechanism of the broadly described anti-inflammatory activity of pseudopterosin by demonstrating a concentration-dependent inhibition of the NF-κB pathway based on inhibition of p65 and IκB phosphorylation.

NF-κB overexpression maintains cancer stem cell populations in the basal-subtype of breast cancer and plays a crucial role in overall cancer progression [29,49,50,51]. NF-κB activity is involved in epithelial-to-mesenchymal transition (EMT) [52]. Thus, previous studies have approached the inhibition of NF-κB activity in several ways: Gordon et al. suppressed NF-κB transcription in MDA-MB-231 breast cancer cells resulting in reduced osteolysis after tumor cell injection in mice combined with decreased cytokine expression [53]. Furthermore, inhibition of NF-κB activity in human breast cancer cells (MDA-MB-231 and HCC1954) reduced invasiveness and migration [52]. In conclusion, NF-κB activation blockade demonstrates effective reduction in tumor growth and progression. Our study revealed pseudopterosin to efficaciously inhibit NF-κB signaling and subsequent cytokine release in both THP-1 monocytic leukemia cells and MDA-MB-231 breast cancer cells. Furthermore, pseudopterosin has demonstrated the ability to block the inter-cell communication between immune cells and MDA-MB-231 breast cancer cells, a complex interplay presumably important within the tumor microenviromental set-up.

Nuclear receptors like the glucocorticoid receptor α (GR) translocate into the nucleus upon activation and bind the glucocorticoid response element (GRE) enabling the transcription of target genes ultimately resulting in immune suppression. Thus, GR and NF-κB are transcription factors with opposing functions in regulating inflammatory responses. In cancer therapy glucocorticoids are used as a pre-treatment combined with chemotherapy to prevent vomiting and allergic reactions [32,38,54]. However, due to high variability in its expression frequency, divergent cellular functions of GR have been described [2]. For instance, high expression levels not only lead to poor prognosis for ER^−^ breast cancer patients, but are also associated with better outcomes in patients with ER^+^ breast cancer [55]. Suppression of chemotherapy induced apoptosis for example is correlated with high GR expression and poor prognosis [37,55,56]. On the other hand, glucocorticoids can suppress migration, invasion and angiogenesis via down-regulation of IL-6 and IL-8. Furthermore, GR agonism has been shown to induce drug sensitivity and apoptosis in lymphoid cancer and breast cancer [36,37,38].

Interestingly, there is evidence that expression of both transcription factors, NF-κB and GR, are correlated in the context of breast cancer. While NF-κB is up-regulated [25,57], GR over-expression could be confirmed for breast cancer, however, in contrast to NF-κB, GR levels decreased during cancer progression [58]. Furthermore, there is evidence that NF-κB and GR can even physically interact by heterodimerization [35,51]. Glucocorticoids regulate target genes by either positive or negative regulatory mechanisms. Anti-inflammatory effects are mediated via a transcription repressive function (so called transrepressive action) of GR, whereas activation of gene transcription (namely transactivation) results in an undesirable side effect of glucocorticoids including chemoresistance, impaired wound-healing, and skin and muscle atrophy [59,60,61]. A previous study revealed that NF-κB inhibition is likely based on the transrepressive function of GR [1]. Our study confirms GR as putative pharmacological target of pseudopterosins. In conclusion, we hypothesize that the induction of GR activation upon pseudopterosin treatment might be based on GR acting as transrepressive on NF-κB.

As triple-negative breast cancer represents one of the diseases with a high unmet medical need resulting in a low overall survival rate, there is a need for efficacious drug treatment regimens. Our study contributes by elucidating the molecular mode of action of the striking anti-inflammatory effect of the marine diterpene glycosides PsA-D in the context of breast cancer. Thus, we demonstrate the mostly unexplored pharmaceutical potential of pseudopterosins as a promising basis for developing novel cancer treatment strategies. Future studies may include a medicinal chemistry approach to design simplified derivatives of pseudopterosin with improved potency.

## 4. Materials and Methods

### 4.1. Cell Culture and Commercially Available Reagents

TNFα was purchased from Peprotech (Rocky Hill, NJ, USA). MDA-MB-231 breast cancer cells were obtained from European Collection of Authenticated Cell Cultures (Salisbury, UK) and grown in humidified atmosphere containing no CO_2_ in Leibovitz’s L15 medium. Medium was supplemented with 15% FCS (fetal calf serum), 2 mM glutamine, 100 unit’s mL^−1^ penicillin and 100 µg mL^−1^ unit’s streptomycin. THP-1 acute monocytic leukemia cells were purchased from the German Collection of Microorganisms and Cell Culture (Braunschweig, Germany) and cultured in the presence of 5% CO_2_ in RPMI along with 10% FCS, penicillin and streptomycin. This cell line was used as a model for cells derived from the immune system. Medium and antibiotics were purchased from Gibco (Life Technologies, Carlsbad, CA, USA).

### 4.2. Stable Cell Line Generation

MDA-MB-231 breast cancer cells were used to create a stable cell line subsequently named NF-κB-MDA-MB-231 where the expression of a Luciferase reporter gene is under the control of a NF-κB CMV promoter. The vector was purchased from Promega (Madison, WI, USA): pNL3.2.NF-κB-RE[NlucP/NF-κB-RE/Hygro]. Cells were transfected with the nucleofector 2b device from Lonza Group AG (Basel, Switzerland) and the corresponding RCT Cell Line Kit V according to the manufacturer’s protocol. Cells were cultured in DMEM supplemented with 10% FCS, 100 units mL^−1^ penicillin and 100 units mL^−1^ streptomycin. After transfection cells were diluted serially to obtain monoclonal cells. After colony formation hygromycin (Sigma, Munich, Germany) clones were cultivated in the presence of hygromycin.

### 4.3. NF-κB Reportergene Assay

To determine NF-κB activation, cells were seeded with a density of 5 × 10^5^ cells per mL in 384-well plates using the CyBio^®^ pipetting roboter (Analytic Jens AG; Jena, Germany). After 24 h of incubation, cells were treated with different concentrations of PsA-D for 20 min. Afterwards, cells were treated with 1 µg/mL LPS or 6 ng/mL TNFα for 1 h, respectively. Luciferase activity was detected with the NanoGlo Luciferase Assay from Promega. NanoGlo Substrate and buffer were pre-mixed in 1:50 ratio and reagent was added to the wells in a 1:1 ratio and luminescence was determined immediately.

### 4.4. NF-κB and Human Cytokine Magnetic Bead Kit

MDA-MB-231 breast cancer cells were cultured in 10 cm dishes in 1.8 × 10^6^ cells per mL and incubated for 24 h at 37 °C. Before compound treatment medium was changed to serum-free medium. Cells were treated with PsA-D for 15 min, followed by incubation with 1 µg/mL LPS. Afterwards, cells were lysed with the lysis buffer provided in the NF-κB magnetic bead kit from Merck Millipore (Darmstadt, Germany) to obtain phosphorylated proteins from the nucleus. Protein concentration was determined with Bradford reagent (Roth, Karlsruhe; Germany). Samples were diluted to achieve a concentration of 0.8 mg/mL of total proteins. The subsequent protocol was according to manufacturer’s instructions.

MDA-MB-231 breast cancer cells and were seeded in 96-well plates in 4 × 10^5^ cells per mL and MDA-MB-453 in 6 × 10^5^ cells per mL and incubated for 24 h at 37 °C. THP-1 cells were seeded in 4 × 10^5^ cells per mL and after 1 h of incubation differentiated with 10 ng/mL PMA for 24 h. Cells were treated with PsA-D for 20 min and afterwards with 1 µg/mL LPS for 24 h. Supernatant was harvested and stored at −20 °C until measurement of cytokines. The subsequent protocol was performed according to the manufacturer’s instructions with the MAGPIX^®^ Multiplexing System from Merck Millipore (Darmstadt, Germany).

### 4.5. Quantitative Real-Time PCR

To determine cytokine expression levels after PsA-D treatment, the following primers were used (purchased from Eurofins, Ebersberg): IL-6 forward (GGCACTGGCAGAAAACAACC), IL-6 reverse (GCAAGTCTCCTCATTGAATCC) IL-8 forward: (ACTGAGAGTGATTGAGAGTGGAC), IL-8 reverse: (AACCCTCTGCACCCAGTTTTC), TNFα forward: (GCCTGCTGCACTTTGGAGTG), TNFα reverse: (TCGGGGTTCGAGAAGATGAT), MCP-1 forward: (CCCCAGTCACCTGCTGTTAT), MCP-1 reverse: (TGGAATCCTGAACCCACTTC), GAPDH forward: (TGCACCACCAACTGCTTAGC), GAPDH reverse: (GGCATGGACTGTGGTCATGAG), GR forward: (AAAAGAGCAGTGGAAGGACAGCAC) GR reverse: (GGTAGGGGTGAGTTGTGGTAACG). Total RNA was isolated with QIAGEN (Hilden, Germany) RNA Isolation Kit according to manufacturer’s instructions and reverse transcriptase PCR were performed with iScript RT cDNAse Kit from BioRad (Munich, Germany). Real-time PCR was conducted with Quantitect SYBR Green from QIAGEN (Hilden, Germany) based on the following protocol: pre-incubation at 95 °C for 900 s, amplification was performed over 45 cycles (95 °C for 15 s, 55 °C for 25 s and 72 °C for 10 s). No-template controls served as negative control. *C*_T_ values were calculated according to the $2^{-\Delta \Delta C_{T}}$ method [62]. Sample values were normalized to the house-keeping gene GAPDH (glyceraldehyde 3-phosphate dehydrogenase).

### 4.6. Immunofluorecent Staining

MDA-MB-231 breast cancer cells were seeded in 1 × 10^5^ cells per mL and incubated for 24 h. PsA-D or dexamethasone treatment comprised 30 min. Cells were fixed afterwards with −10 °C cold methanol. Cells were made permeable using 0.1% Triton™ X-100. Antibodies were purchased from Santa Cruz Biotechnology (Dallas, TX, USA): primary antibody (sc-8992 GR (H-300)) incubated 1:50 for 24 h overnight at 4 °C and secondary antibody (sc-2012 IgG-FITC (fluorescein isothiocyanate)) was incubated 1:100 for 2.5 h at room temperature. Cells were washed three times with PBS following each incubation step. For staining, cell nuclei 4′,6-Diamidin-2-phenylindol (DAPI, Sigma) was incubated for 5 min at room temperature at a concentration of 3 µM and washed three times with PBS for 5 min.

Quantification of immunofluorescence intensity was achieved with ImageJ (v1.51k). The shape of the cells was outlined and the area, mean gray fluorescence value and integrated density measured. Several background readings were also measured. The “total corrected cellular fluorescence” (=TCCF) was calculated according to following formula: integrated density—(area of selected cell x mean fluorescence of background readings) [63]. Values of GFP staining were subtracted by values of DAPI staining to obtain cytoplasmic TCCF.

### 4.7. Conditioned Medium (CM) from Tumor Cells

MDA-MB-231 or THP-1 cells were cultured until 70–90% confluency. 1 × 10^6^ cells were counted and transferred into a 25 cm^2^ flask. Cells were either stimulated with 1 µg/mL LPS or without LPS as a negative control. Supernatant was collected after 24 h, centrifuged and sterile filtered. Conditioned medium was stored at −80 °C. MDA-MB-231 or THP-1 cells were seeded at 1 × 10^6^ cells per mL in 6-well plates and incubated for 24 h. PsA-D was added at a concentration of 30 µM for 20 min followed by 25 volume percentage of tumor-conditioned medium for 5 h. Cells were then harvested and RNA isolated for further analysis in real-time PCR.

### 4.8. Knock-Down Studies

TLR4 siRNA s14194 and Silencer^®^ Select Negative Control No. 2 siRNA was purchased from Life Technologies (Darmstadt, Germany). Glucocorticoid receptor (GR) siRNA was purchased from Santa Cruz Biotechnology (Dallas, TX, USA). SiRNA transfection (2 µM of siRNA) was performed using Lipofectamine3000 from Invitrogen (Carlsbad, CA, USA) according to manufacturer’s protocol. 

### 4.9. GR Reportergene Assay

Reportergene assay based on non-human stable cells containing constitutive high-level expression of full-length human GR (NR3C1) were purchased from Indigo Biosciences (State College, PA, USA). Assay was performed according to manufacturer’s instructions. PsA-D was added to cells according to the agonist assay described in the protocol and incubated for 24 h at 37 °C.

### 4.10. Preparation of PsA-D Mixture

*A. elisabethae* was collected from South Bimini Island, The Bahamas, was dried and extracted in EtOAc/MeOH (1:1) for 48 h. The crude extract was subjected to silica gel chromatography eluting with hexanes and EtOAc to afford a mixture of PsA-D. The ratio was determined to be 85:5:5:5 (PsA:B:C:D) by LC-MS analysis.

### 4.11. Statistical Analysis

Obtained data represent at least three independent experiments. Error bars show +SEM of the means of triplicate values. Statistical analysis was calculated using one-way-ANOVA followed by Dunnett's multiple comparisons test. When groups were compared with a control and/or comparison of mean values of only two groups, an unpaired student’s *t*-Test was applied. *p* < 0.05 was chosen to define statistically significant difference. Figures and data analysis were generated with Graphpad Prism v. 6.07 (Graphpad Software, San Diego, CA, USA).

## Figures and Tables

**Table 1:** Inhibition of cytokine release in THP-1 monocytic leukemia and MDA-MB-231 triple negative breast cancer. THP-1 cells were treated with 10 ng PMA (phorbol 12-myristate 13-acetate) for 24 h to induce differentiation. Cytokine amounts were analyzed in supernatants after a 24-h incubation time. Total amounts of cytokines (pg/mL) were calculated according to a standard concentration curve. No treatment serves as a control. % inhibition reflects the percentage of cytokines reduced by PsA-D treatment. Standard deviation was calculated for amounts of cytokines (±SD); *n* = 3. TNF: tumor necrosis factor alpha; IL: interleukin; MCP: monocyte chemotactic protein 1.

MDA-MB-231	Control (pg/mL)	+LPS 1 µg/mL	+PsA-D 30 µM	*p*-Value	% Inhibition
IL-6	1626.3 ± 144	4666.7 ± 307	2874.8 ± 610	<0.0002	38.3
TNFα	1.9 ± 0.6	29.1 ± 5.5	7.17 ± 3.4	<0.0005	75.3
MCP-1	325.3 ± 260	1625.6 ± 540.6	241.3 ± 100.9	0.0082	85.2
**THP-1**	**Control (pg/mL)**	**+LPS 1 µg/mL**	**+PsA-D 30 µM**	***p*-Value**	**% Inhibition**
IL-6	2.8 ± 1	66.7 ± 9.8	33 ± 1.98	0.0089	50.0
TNFα	13.4 ± 4.5	439.4 ± 28	232.0 ± 100	0.1138	47.2
MCP-1	182.9 ± 65.3	4436.7 ± 2098	1208.9 ± 762.3	0.0552	72.8

## Supplementary Materials

The following are available online at www.mdpi.com/1660-3397/15/9/262/s1, Figure S1: Pseudopterosin inhibits activation of NF-κB after two different stimuli, Figure S2: Pseudopterosin blocked phosphorylation of p65 and IkBα after TNFα stimulation; Table S1: Inhibition of cytokine release in MDA-MB-453 triple negative breast cancer cells.

## References

[1] Moutsatsou P., Papavassiliou A.G. (2008). The glucocorticoid receptor signalling in breast cancer. J. Cell. Mol. Med..

[2] Belova L., Delgado B., Kocherginsky M., Melhem A., Olopade O., Conzen S. (2015). Glucocorticoid receptor expression in breast cancer associates with older patient age. Breast Cancer Res. Treat..

[3] Pal S.K., Childs B.H., Pegram M. (2011). Triple negative breast cancer: Unmet medical needs. Breast Cancer Res. Treat..

[4] Abad M.J., Bermejo P. (2001). Bioactive natural products from marine sources. Stud. Nat. Prod. Chem..

[5] Berrué F., McCulloch M.W.B., Kerr R.G. (2011). Marine diterpene glycosides. Bioorg. Med. Chem..

[6] Mayer A.M.S., Jacobson P.B., Fenical W., Jacobs R.S., Glaser K.B. (1998). Pharmacological characterization of the pseudopterosins: Novel anti-inflammatory natural products isolated from the Caribbean soft coral, *Pseudopterogorgia elisabethae*. Life Sci..

[7] Correa H., Valenzuela A.L., Ospina L.F., Duque C. (2009). Anti-inflammatory effects of the gorgonian *Pseudopterogorgia elisabethae* collected at the Islands of Providencia and San Andrés (SW Caribbean). J. Inflamm. Lond..

[8] Ata A., Kerr R.G., Moya C.E., Jacobs R.S. (2003). Identification of anti-inflammatory diterpenes from the marine gorgonian *Pseudopterogorgia elisabethae*. Tetrahedron.

[9] Look S.A., Fenical W., Matsumoto G.K., Clardy J. (1986). The pseudopterosins: A new class of antiinflammatory and analgesic diterpene pentosides from the marine sea whip *Pseudopterogorgia elisabethae* (Octocorallia). J. Org. Chem..

[10] Look S.A., Fenical W., Jacobs R.S., Clardy J. (1986). The pseudopterosins: Anti-inflammatory and analgesic natural products from the sea whip *Pseudopterogorgia elisabethae*. Proc. Natl. Acad. Sci. USA.

[11] Caplan S.L., Zheng B., Dawson-Scully K., White C.A., West L.M. (2016). Pseudopterosin A: Protection of synaptic function and potential as a neuromodulatory agent. Mar. Drugs.

[12] Newman D.J., Cragg G.M. (2004). Marine natural products and related compounds in clinical and advanced preclinical trials. J. Nat. Prod..

[13] Mayer A.M.S., Glaser K.B., Cuevas C., Jacobs R.S., Kem W., Little R.D., Mcintosh J.M., Newman D.J., Potts B.C., Shuster D.E. (2010). The odyssey of marine pharmaceuticals: A current pipeline perspective. Trends Pharmacol. Sci..

[14] Mayer A.M.S., Rodriguez A.D., Berlinck R.G.S., Hamann M.T. (2008). Marine pharmacology in 2003–4: Marine compounds with anthelminthic, antibacterial, anticoagulant, antifungal, anti-inflammatory, antimalarial, antiplatelet, antiprotozoal, antituberculosis, and antiviral activities; affecting the cardiovascular, immune. Comp. Biochem. Physiol. C Toxicol. Pharmakol..

[15] Moya C.E., Jacobs R.S. (2006). Pseudopterosin A inhibits phagocytosis and alters intracellular calcium turnover in a pertussis toxin sensitive site in *Tetrahymena thermophila*. Comp. Biochem. Physiol. C Toxicol. Pharmacol..

[16] Rodríguez I.I., Shi Y.P., García O.J., Rodríguez A.D., Mayer A.M.S., Sánchez J.A., Ortega-Barria E., González J. (2004). New pseudopterosin and seco-pseudopterosin diterpene glycosides from two Colombian isolates of *Pseudopterogorgia elisabethae* and their diverse biological activities. J. Nat. Prod..

[17] Badr C., Niers J.M., Tjon-Kon-Fat L.-A., Noske D.P., Wurdinger T., Tannous B. (2009). Real-time monitoring of NF-kappaB activity in cultured cells and in animal models. Mol. Imaging.

[18] Kawai T., Akira S. (2010). The role of pattern-recognition receptors in innate immunity: Update on Toll-like receptors. Nat. Immunol..

[19] Ramage L. (2003). Expression of Pro-Inflammatory Proteins in the Lung Epithelial Cell Line A549, in Response to Cytokine, and Environmental Particle Exposure. Ph.D. Thesis.

[20] Blank V., Kourilsky P., Israel A., Publishers E.S. (1992). NF-kB and related proteins: Rel/dorsal homologies meet ankyrin-like repeats. Trends Biochem. Sci..

[21] Baeuerle P.A., Baltimore D. (1988). Activation of DNA-binding activity in an apparently cytoplasmic precursor of the NF-kappa B transcription factor. Cell.

[22] Balkwill F. (2006). TNF-alpha in promotion and progression of cancer. Cancer Metastasis Rev..

[23] Zhang L., Blackwell K., Altaeva A., Shi Z., Habelhah H. (2010). TRAF2 phosphorylation promotes NF-κB-dependent gene expression and inhibits oxidative stress-induced cell death. Mol. Biol. Cell.

[24] Perkins N.D. (2007). Integrating cell-signalling pathways with NF-kappaB and IKK function. Nat. Rev. Mol. Cell Biol..

[25] Cai C., Yao Z. (2006). Activation of NF-κB in human breast cancer and its role in cell proliferation and progression. Chin. J. Clin. Oncol..

[26] Zhao X., Sun X., Gao F., Luo J., Sun Z. (2011). Effects of ulinastatin and docataxel on breast tumor growth and expression of IL-6, IL-8, and TNF-α. J. Exp. Clin. Cancer Res..

[27] Park M., Hong J. (2016). Roles of NF-κB in cancer and inflammatory diseases and their therapeutic approaches. Cells.

[28] Shostak K., Chariot A. (2011). NF-κB, stem cells and breast cancer: The links get stronger. Breast Cancer Res..

[29] Becker-Weimann S., Xiong G., Furuta S., Han J., Kuhn I., Akavia U.-D., Pe’er D., Bissell M.J., Xu R. (2013). NFkB disrupts tissue polarity in 3D by preventing integration of microenvironmental signals. Oncotarget.

[30] Bissell M.J., Radisky D. (2001). Putting tumors in context. Nat. Rev. Cancer.

[31] Mestdagt M., Polette M., Buttice G., Noël A., Ueda A., Foidart J. (2006). Transactivation of MCP-1/CCL2 by beta-catenin/TCF-4 in human breast cancer cells. Int. J. Cancer.

[32] Keith B.D. (2008). Systematic review of the clinical effect of glucocorticoids on nonhematologic malignancy. BMC Cancer.

[33] Skor M., Wonder E., Kocherginsky M., Goyal A., Hall B., Cai Y., Conzen S. (2012). Glucocorticoid receptor antagonsims as a novel therapy for triple-negative breast cancer. Clin. Cancer Res..

[34] Mitre-Aguilar I.B., Cabrera-Quintero A.J., Zentella-Dehesa A. (2015). Genomic and non-genomic effects of glucocorticoids: Implications for breast cancer. Int. J. Clin. Exp. Pathol..

[35] McKay L.I., Cidlowski J.A. (1999). Molecular control of immune/inflammatory responses: Interactions between nuclear factor-κB and steroid receptor-signaling pathways. Endocr. Rev..

[36] Schmidt S., Rainer J., Ploner C., Presul E., RimL S., Kofler R. (2004). Glucocorticoid-induced apoptosis and glucocorticoid resistance: Molecular mechanisms and clinical relevance. Cell Death Differ..

[37] Lin K.T., Wang L.H. (2016). New dimension of glucocorticoids in cancer treatment. Steroids.

[38] Buxant F., Kindt N., Laurent G., Noel J., Saussez S. (2015). Antiproliferative effect of dexamethasone in the MCF-7 breast cancer cell line. Mol. Med. Rep..

[39] Mehmeti M., Allaoui R., Bergenfelz C., Saal L.H., Ethier S.P., Johansson M.E., Jirström K., Leandersson K. (2015). Expression of functional toll like receptor 4 in estrogen receptor/progesterone receptor-negative breast cancer. Breast Cancer Res..

[40] Akira S., Uematsu S., Takeuchi O. (2006). pathogen recognition and innate immunity. Cell.

[41] Tak P.P., Firestein G.S. (2001). NF-κB: A key role in inflammatory diseases. J. Clin. Investig..

[42] Pahl H.L. (1999). Activators and target genes of Rel/NF-κB transcription factors. Oncogene.

[43] Kumar A., Takada Y., Boriek A., Aggarwal B. (2004). Nuclear factor-κB: Its role in health and disease. J. Mol. Med..

[44] Schütze S., Potthoff K., Machleidt T. (1992). TNF activates NF-KB by phosphatidylcholine-specific C-induced “Acidic” sphingomyelin breakdown. Cell.

[45] Miyake K. (2004). Innate recognition of lipopolysaccharide by Toll-like receptor 4-MD-2. Trends Microbiol..

[46] Wajant H., Scheurich P. (2011). TNFR1-induced activation of the classical NF-KB pathway. FEBS J..

[47] Correa H., Aristizabal F., Duque C., Kerr R. (2011). Cytotoxic and antimicrobial activity of pseudopterosins and seco-pseudopterosins isolated from the octocoral *Pseudopterogorgia elisabethae* of san andrés and providencia islands (Southwest Caribbean Sea). Mar. Drugs.

[48] Ata A., Win H.Y., Holt D., Holloway P., Segstro E.P., Jayatilake G.S. (2004). New antibacterial diterpenes from *Pseudopterogorgia elisabethae*. Helv. Chim. Acta.

[49] Yamamoto M., Taguchi Y., Ito-kureha T., Semba K., Yamaguchi N., Inoue J. (2013). NF-κB non-cell-autonomously regulates cancer stem cell populations in the basal-like breast cancer subtype. Nat. Commun..

[50] Yamaguchi N., Ito T., Azuma S., Ito E., Honma R., Yanagisawa Y., Nishikawa A., Kawamura M., Imai J., Watanabe S. (2009). Constitutive activation of nuclear factor-kappaB is preferentially involved in the proliferation of basal-like subtype breast cancer cell lines. Cancer Sci..

[51] Ling J., Kumar R. (2012). Crosstalk between NFkB and glucocorticoid signaling: A potential target of breast cancer therapy. Cancer Lett..

[52] Pires B.R.B., Mencalha A.L., Ferreira G.M., de Souza W.F., Morgado-Díaz J.A., Maia A.M., Corrêa S., Abdelhay E.S.F.W. (2017). NF-kappaB is involved in the regulation of EMT genes in breast cancer cells. PLoS ONE.

[53] Gordon A.H., O’Keefe R.J., Schwarz E.M., Rosier R.N., Puzas J.E. (2005). Nuclear factor-kB-dependent mechanisms in breast cancer cells regulate tumor burden and osteolysis in bone. Am. Assoc. Cancer Res..

[54] Rutz H.P. (2002). Effects of corticosteroid use on treatment of solid tumours. Lancet.

[55] Pan D., Kocherginsky M., Conzen S.D. (2011). Activation of the glucocorticoid receptor is associated with poor prognosis in estrogen receptor-negative breast cancer. Cancer Res..

[56] Mondal S.K., Jinka S., Pal K., Nelli S., Dutta S.K., Wang E., Ahmad A., AlKharfy K.M., Mukhopadhyay D., Banerjee R. (2016). Glucocorticoid receptor-targeted liposomal codelivery of lipophilic drug and Anti-Hsp90 gene: Strategy to induce drug-sensitivity, EMT-reversal, and reduced malignancy in aggressive tumors. Mol. Pharm..

[57] Radisky D.C., Bissell M.J. (2007). NF-kappaB links oestrogen receptor signalling and EMT. Nat. Cell Biol..

[58] Abduljabbar R., Negm O.H., Lai C.-F., Jerjees D.A., Al-Kaabi M., Hamed M.R., Tighe P.J., Buluwela L., Mukherjee A., Green A.R. (2015). Clinical and biological significance of glucocorticoid receptor (GR) expression in breast cancer. Breast Cancer Res. Treat..

[59] Chrousos G.P., Kino T. (2005). Intracellular glucocorticoid signaling: A formerly simple system turns stochastic. Sci. Signal..

[60] De Bosscher K., Vanden Berghe W., Haegeman G. (2003). The interplay between the glucocorticoid receptor and nuclear factor-kB or activator protein-1: Molecular mechanisms for gene repression. Endocr. Rev..

[61] Kino T., De Martino M.U., Charmandari E., Mirani M., Chrousos G.P. (2003). Tissue glucocorticoid resistance/hypersensitivity syndromes. J. Steroid Biochem. Mol. Biol..

[62] Livak K.J., Schmittgen T.D. (2001). Analysis of relative gene expression data using real-time quantitative PCR and $2^{-\Delta \Delta C_{T}}$ method. Methods.

[63] McCloy R.A., Rogers S., Caldon C.E., Lorca T., Castro A., Burgess A. (2014). Partial inhibition of Cdk1 in G2 phase overrides the SAC and decouples mitotic events. Cell Cycle.

