# Supplementary material for: The Marine Natural Product Pseudopterosin Blocks Cytokine Release of Triple-Negative Breast Cancer and Monocytic Leukemia Cells by Inhibiting NF-κB Signaling
Source: Mar Drugs. 2017 Aug 23;15(9):262. doi: 10.3390/md15090262 (PMC5618401; doi:10.3390/md15090262)
Supplement: Supplementary file 1 [file marinedrugs-15-00262-s001.pdf]

Supplement

Figure S1. Pseudopterosin inhibits activation of NF-κB after two different stimuli. Cells are stably transfected with a NF-κB-Luc reporter gene. PsA-D treatment was performed for 20 minutes following 1 µg/ml LPS or 6 ng TNFα incubation for 1 hour. Error bars were calculated using +SEM; n=3. Three stars show a significance of $p<0.001$.

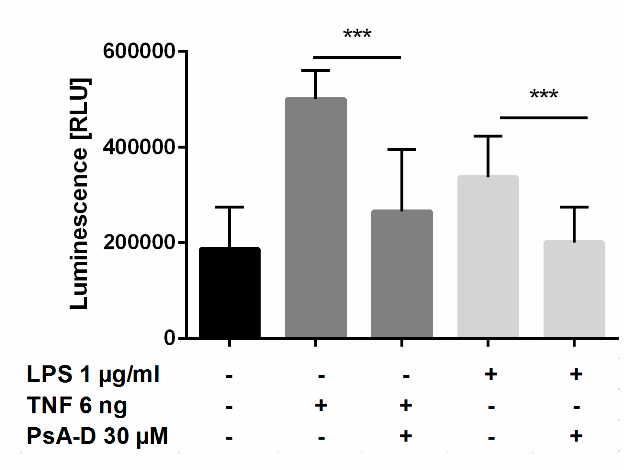

Figure S2. PsA-D blocked phosphorylation of NF-kB/p65 and IkBa. MDA-MB-231 cells were seeded in a 10 cm dish. Treatment with 30 µM PsA-D for 15 minutes was followed with treatment of 6 ng TNFαfor 15 minutes. Cells were lysed and protein concentration was measured with Bradford Reagent. A total protein amount of 0.8 mg/ml was used. Control cells were treated with DMSO in the same amounts as PsA-D. Error bars were calculated using +SEM; n=3. P-values of three stars show a significance of p<0,001.

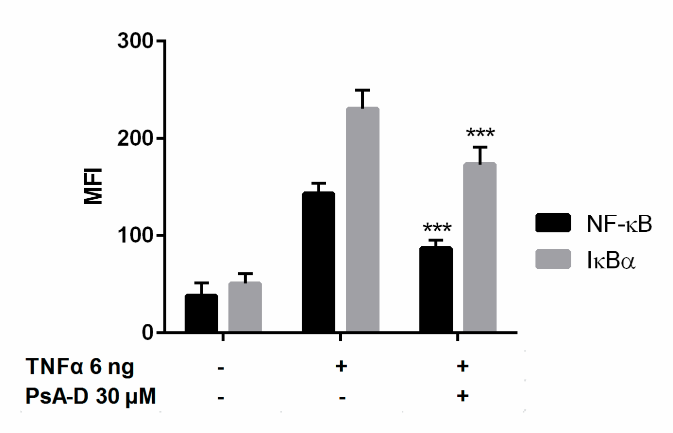

Table S1. Inhibition of cytokine release in MDA-MB-453 triple negative breast cancer cells. MDA-MB-453 cells were seeded at a density of $6*10^6$ cells per ml. 30 μM PsA-D was incubated for 20 minutes followed by 20 ng/ml TNFα. Cytokine amounts were analyzed in supernatants after 24 hours incubation time. No treatment serves as a control. Values were normalized to TNFα treatment and set to 100 % ±SD. % inhibition reflects the percentage of the amount of cytokines reduced by PsA-D treatment compared to TNFα. P-values were analyzed according to student's t-test; n=3.

MDA-MB-453	Control %	+TNFα %	+PsA-D %	p-value	% Inhibition
IL-8	4,7 (±5,5)	100,5 (±24,9)	51,7 (±14,0)	0,012	48,9
MCP-1	17,3 (±11,6)	97,4 (±7,9)	87,2 (±14,1)	0,43	10,2
